# Effects of Regular Exercise on Peripheral Brain-Derived Neurotrophic Factor in Neurological and Non-Neurological Populations: A Meta-Analysis with Meta-Regression

**DOI:** 10.3390/brainsci16010039

**Published:** 2025-12-27

**Authors:** Mesut Süleymanoğulları, Aslıhan Tekin, Halil İbrahim Ceylan, Gökhan Bayraktar, Tolga Altuğ, Raul Ioan Muntean, Cemre Didem Eyipınar

**Affiliations:** 1 Department of Physical Education and Sports Teaching, Faculty of Sport Sciences, Agri Ibrahim Cecen University, Agri 04100, Turkey; msuleymanogullari@agri.edu.tr (M.S.); astekin@agri.edu.tr (A.T.); gbayraktar@agri.edu.tr (G.B.); taltug@agri.edu.tr (T.A.); 2 Physical Education of Sports Teaching Department, Faculty of Sports Sciences, Ataturk University, Erzurum 25240, Turkey; 3 Department of Physical Education and Sport, Faculty of Law and Social Sciences, University “1 Decembrie 1918” of Alba Iulia, 510009 Alba Iulia, Romania; 4 Department of Physical Education and Sports Teaching, Faculty of Sport Sciences, Gaziantep University, Gaziantep 27410, Turkey; cemreeyipinar@gantep.edu.tr

**Keywords:** brain-derived neurotrophic factor, exercise, neuroplasticity, serum biomarkers, cognitive rehabilitation, mental health

## Abstract

**Background**: Brain-derived neurotrophic factor (BDNF) is a key regulator of neuroplasticity, cognitive function, and mental health. Exercise is proposed as a non-pharmacological strategy to enhance BDNF; however, findings across neurological and non-neurological disorders remain inconsistent, and the influence of exercise type or dose-related parameters remains unclear. **Objective**: This meta-analysis evaluated the effects of exercise interventions on peripheral BDNF levels in individuals with neurological (e.g., multiple sclerosis, cognitive impairment, schizophrenia, depression) and non-neurological (e.g., obesity, type 2 diabetes, cancer) disorders, and examined whether outcomes varied by disease category, exercise modality, or dose. **Methods**: A systematic search of Web of Science, PubMed, ScienceDirect, Scopus, and Cochrane was conducted up to 1 October 2025. Eligible randomized controlled trials (RCTs) and the quality of evidence were assessed using the PEDro scale and the GRADE approach, respectively. Random-effects models were applied, with subgroup analyses (neurological vs. non-neurological; exercise type; duration and assay type), meta-regressions (duration, frequency, session length), and publication bias tests (funnel plot, Begg’s test, Egger’s regression, and trim-and-fill). **Results**: Nineteen RCTs, including 850 participants, were analyzed. According to low-quality evidence, exercise significantly increased peripheral BDNF (SMD = 1.03, 95% CI: [0.56–1.49, *p* < 0.0001). Effects did not differ significantly between neurological (SMD = 0.91, 95% CI: 0.31–1.50) and non-neurological (SMD = 1.23, 95% CI: 0.47–1.99) conditions (Q (1) = 0.44, *p* = 0.51). Subgroup analyses revealed significant improvements for resistance exercise (SMD = 1.57, 95% CI: 0.91–2.23), followed by aerobic (SMD = 1.44, 95% CI: 0.36–2.52) and combined exercise (SMD = 0.55, 95% CI: 0.21–0.89). Meta-regressions showed no moderating effects of duration (β = 0.0101, *p* = 0.834), weekly frequency (β = 0.1464, *p* = 0.648), minutes per session (β = −0.0124, *p* = 0.233) or total weekly minutes (β = 0.0005, *p* = 0.919) apart from age and baseline BDNF level factors (β = 0.0348, *p* = 0.020; β = −0.035, *p* = 0.0258). Publication bias tests indicated minimal publication bias, with adjusted effects remaining robust. **Conclusions**: Exercise interventions have been shown to increase peripheral BDNF significantly across diverse clinical populations. In particular, resistance and aerobic exercise protocols accounted for the exploratory component, whereas simple dose-related factors did not explain the variability. These findings are consistent with the biological plausibility of exercise-induced neuroplasticity and underscore the need for larger, pre-registered RCTs with harmonized biomarker protocols to strengthen clinical translation. However, the certainty of evidence is limited by small sample sizes and the frequent lack of blinding of participants and assessors across included trials.

## 1. Introduction

Brain-derived neurotrophic factor (BDNF) is the most abundant neurotrophin in the human central nervous system (CNS) [1]. It is predominantly expressed in the prefrontal cortex, amygdala, hippocampus, and other limbic regions [2]. As a key growth factor, BDNF regulates neurogenesis, gliogenesis, and neurite outgrowth [3,4] while enhancing dendritic growth in an activity-dependent manner [5].

A wealth of evidence suggests that exercise enhances both cognition [6,7] and mood [8,9,10], with BDNF activity likely serving as a crucial mediator [11,12,13,14]. Non-quantitative reviews from animal and human studies suggest that BDNF levels rise following exercise in rodents and acute or programmed aerobic exercise in humans [15,16]. Consequently, BDNF is widely recognized as a primary molecular mechanism underlying exercise-induced improvements in executive function [14,17,18].

Numerous studies have linked exercise-induced BDNF changes to various diseases, including multiple sclerosis (MS), where the immune system attacks myelin in the CNS [19,20,21], obesity affecting neurological health [22,23], cancer associated with psychological stress and depression [24], and neurodegenerative conditions like dementia, depression, mental illnesses, and cognitive impairment [25,26,27,28,29,30], alongside schizophrenia [31,32] and type 2 diabetes with neurological impacts [33]. However, the heterogeneity across these studies—due to diverse participant diseases, exercise types, and durations—limits the generalizability of findings. Previous reviews lack a quantitative synthesis to address this variability [34], underscoring a critical gap in understanding how exercise interventions differentially affect BDNF levels.

Although several meta-analyses have examined the influence of exercise on peripheral BDNF levels, they differ substantially in scope, population selection, methodological criteria, and conceptual purpose, leaving significant gaps that the existing literature has not yet addressed. For instance, Szuhany et al. [35] conducted the first quantitative synthesis of human exercise–BDNF responses (29 studies; N = 1111) and reported that a single acute exercise session produced a moderate increase in circulating BDNF (Hedges’ g = 0.46), while chronic exercise alone elicited only a small effect on resting BDNF (g = 0.27), and regular training slightly amplified the acute response (g = 0.59). However, their dataset consisted primarily of healthy adults and heterogeneous exercise protocols, which prevented conclusions about differences across clinical populations or specific metabolic, neurological, or psychiatric conditions.

Dinoff et al. [36] expanded the chronic-exercise evidence base by synthesizing 29 studies (N = 910), but their eligibility criteria were deliberately restrictive: interventions lasting ≥2 weeks and exclusion of all diseased populations—including metabolic diseases (e.g., diabetes, obesity), neurological disorders (e.g., Parkinson’s disease, multiple sclerosis), and psychiatric conditions (e.g., depression, schizophrenia)—as well as children and studies with co-interventions capable of influencing BDNF. Because all acute-exercise trials and all clinical samples were excluded, their conclusions apply only to chronic training adaptations in non-clinical adult populations. Despite reporting a slight pooled increase in resting BDNF following chronic exercise (SMD = 0.39), this design precludes generalization to populations in which BDNF dysregulation is most clinically relevant.

More recently, Ceylan et al. [37] conducted the first systematic review and meta-analysis dedicated exclusively to individuals with obesity, synthesizing 16 studies and 23 trials (N = 675) and demonstrating that acute exercise increased circulating BDNF levels (ES = 1.25). In contrast, chronic exercise did not yield a statistically significant change despite a small pooled effect (ES = 0.49, *p* = 0.089). Although foundational for understanding BDNF responses in obesity—a metabolic condition consistently associated with reduced basal BDNF—the single-disease focus prevents broader inference across other chronic disorders, and the limited number of obesity-specific trials restricts detailed moderator analyses.

Consequently, despite these three major meta-analyses, the field still lacks a single integrated synthesis that focuses exclusively on chronic exercise, includes a wide range of clinical populations—such as neurological, psychiatric, metabolic, cardiovascular, musculoskeletal, oncologic, and renal disorders—and systematically evaluates key exercise-related moderators, including intervention duration, weekly frequency, session duration, total weekly training volume, and participant characteristics relevant to chronic training effects.

To address these persistent gaps, the present meta-analysis incorporates the largest and most clinically diverse dataset to date, including 19 randomized controlled trials and 850 participants spanning a broad spectrum of health conditions. In contrast to prior syntheses—such as Szuhany et al. [35], which primarily included healthy adults; Dinoff et al. [36], which excluded all clinical populations and all acute-exercise studies; and Ceylan et al. [37], which focused exclusively on obesity—the current study evaluates chronic exercise-induced BDNF responses across the full clinical landscape using standardized methodological criteria. Additionally, by applying structured moderator analyses and multivariate meta-regression, the present study identifies the independent contributions of exercise modality, intensity, volume, duration, frequency, and participant characteristics (e.g., age), thereby clarifying how exercise parameters interact with disease physiology to shape BDNF adaptations. Accordingly, we employed meta-analytic methods to quantitatively synthesize data from randomized controlled trials (RCTs) [34], leveraging their strength in minimizing bias and ensuring internal validity. This approach aims to clarify whether exercise-induced alterations in serum/plasma BDNF levels vary across disease conditions and intervention modalities, thereby establishing a stronger, more clinically relevant foundation for future research.

## 2. Methods and Analysis

### 2.1. Study Design

**Figure 1:**
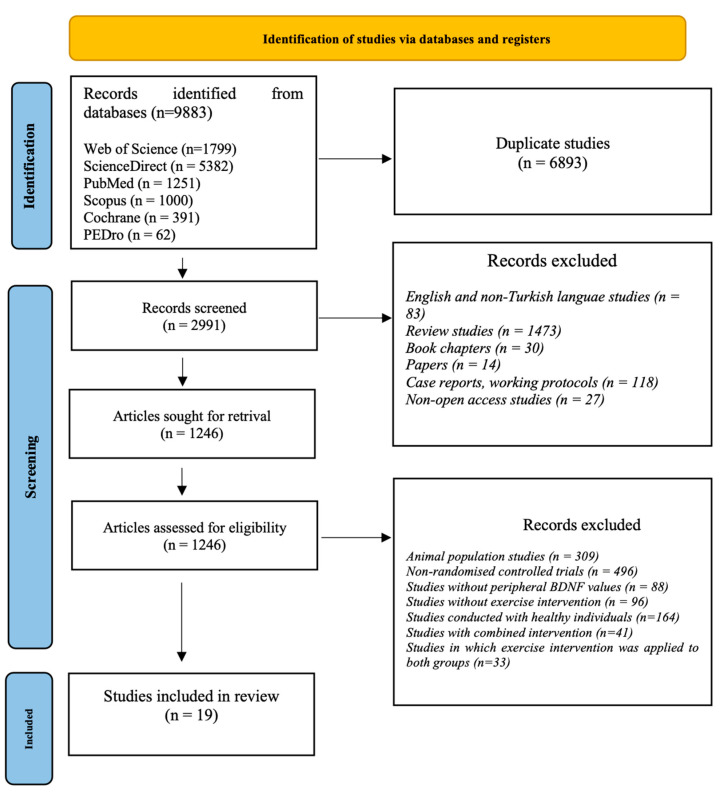
PRISMA Flow Diagram.

The preparation and reporting of this review were conducted according to the Preferred Reporting Items for Systematic Reviews and Meta-Analyses (PRISMA) guidelines [38], with a PRISMA flow diagram (Figure 1) detailing the study selection process and registered in the International Prospective Register of Systematic Reviews (PROSPERO) under registration number CRD42024525971 on 30 March 2024.

### 2.2. Types of Studies

This meta-analysis included RCTs examining the effects of exercise interventions on BDNF levels in individuals with diseases such as multiple sclerosis, obesity, cancer, dementia, depression, cardiovascular diseases, mental disorders, schizophrenia, cognitive impairment, hemodialysis patients with depressive symptoms, type 2 diabetes, hemiplegia after stroke, severe mental illness, and mild cognitive impairment. Only studies with full-text availability in English were included.

### 2.3. Electronic Database Search

Electronic databases were systematically searched from inception until 1 October 2025, using combined search terms including ‘brain-derived neurotrophic factor’, ‘BDNF’, ‘exercise’, ‘physical activity’, ‘sport’, ‘randomised controlled trial’, ‘RCT’, and ‘disease’ in Web of Science, PubMed, ScienceDirect, Scopus, Cochrane, and the Physiotherapy Evidence Database (PEDro) [39]. Boolean operators (e.g., AND, OR) were used, and searches were limited to English-language publications. Additionally, reference sections of identified articles and relevant reviews were manually examined to identify studies not captured by the initial search.

### 2.4. Outcome Measurements

The primary outcome of interest was the change in serum or plasma BDNF levels in response to regular exercise interventions, which, in the included studies, was quantified using established biological techniques such as enzyme-linked immunosorbent assay (ELISA), routine chemistry analyzers, Western blot, quantitative polymerase chain reaction (qPCR), histological examination, immunofluorescent staining, and flow cytometry.

### 2.5. Eligibility Criteria

Four authors (MS, AT, TA, CDE) independently evaluated each article; preliminary screening was based on titles and abstracts, followed by full-text review during the secondary screening. Disagreements were resolved through discussion with a reviewer (AÖ). Articles were included based on the following eligibility criteria: (1) human studies involving people of all ages, including men and women, (2) populations with various diseases, (3) BDNF measured in serum or plasma as the primary outcome, (4) inclusion of a regular exercise intervention, and (5) use of RCTs. Studies were excluded if they were: (1) animal studies, (2) lacking BDNF measurement in serum or plasma, (3) review articles, (4) involving healthy populations, (5) without an exercise intervention, (6) acute studies, or (7) not RCTs.

### 2.6. Quality Assessment

Studies were appraised for methodological quality and risk of bias using the Physiotherapy Evidence Database (PEDro) criteria [40], a reliable and valid checklist comprising 11 items: (1) eligibility criteria, (2) random allocation, (3) concealed allocation, (4) baseline comparability, (5) masked participants, (6) masked therapists, (7) masked assessors, (8) adequate follow-up, (9) intention-to-treat analysis, (10) between-group comparison, and (11) point estimates and variability. Note that the eligibility criteria item does not contribute to the total score; therefore, the score ranges from 0 to 10. Studies were categorized as “poor” (score < 4), “fair” (4–5), “good” (6–8), or “excellent” (9–10) quality [41]. Across trials, PEDro items related to blinding (participants, therapists, assessors) were rarely fulfilled, and most studies enrolled relatively small samples. These features indicate a nontrivial risk of bias and imprecision, which should be considered when interpreting pooled effects. Two authors (M.S., A.T.) independently assessed the quality of each study; disagreements were resolved through discussion with a reviewer (A.Ö.). These authors also used the Grading of Recommendations Assessment, Development and Evaluation (GRADE; www.gradepro.org) system to assess the level of evidence for the meta-analysis’s primary outcomes. The PEDro scale results, reflecting the internal validity and methodological quality of the included studies, are summarized in Table 1, and the GRADE scale results, which assess the quality of the meta-analysis’s main finding, are summarized in Table 2.

The data in Table 1 show that the mean publication quality score for the studies is approximately 7, which is at a good level.

The level of evidence (GRADE) was low for the main outcome, as presented in Table 2. The certainty of evidence for each outcome was assessed using the GRADE approach, which considers risk of bias, inconsistency, indirectness, imprecision, and publication bias. Evidence for BDNF levels was downgraded to low quality due to the risk of bias in the included studies and heterogeneity.

## 3. Data Collection and Analysis

### 3.1. Study Selection: Publication

A two-stage screening approach was employed: titles and abstracts were initially screened, followed by full-text screening. Population, intervention, comparison, outcome, and study design were used to guide the inclusion of studies screened. When study-level information was unavailable during the initial screening phase, full-text access was provided. The study selection process was reported in accordance with the PRISMA 2020 flow diagram (Figure 1).

Nineteen original articles were included for analysis. The search strategy yielded 9883 articles, of which 6893 were duplicates, leaving 2991 for screening. A total of 1745 articles were excluded based on title and abstract. One thousand two hundred forty-six articles met the eligibility criteria, and full-text copies were retrieved. Upon full-text review, an additional 1227 articles were excluded for failing to meet all eligibility criteria, leaving 19 for inclusion in the analysis.

### 3.2. Data Extraction

Study data were extracted using a standardized form that included the first author, publication year, sample sizes of the experimental and control groups, study design, participant gender and age, intervention scheme (session duration, frequency, and assay type), and outcome measures. Peripheral BDNF levels (means and standard deviations) were extracted from studies reporting these data. When data were reported in alternative formats, the corresponding authors were contacted to obtain numerical values. If unavailable, data were extracted from graphs using WebPlotDigitizer software (https://automeris.io/WebPlotDigitizer/ accessed on 1 May 2025). Data from the studies by Gravesteijn et al. [21], Tsai et al. [29], and Dai et al. [31] were obtained using WebPlotDigitizer.

Following duplicate removal, four reviewers (M.S., A.T., T.A., C.D.E.) independently screened articles identified in the systematic review. Initial screening involved reviewing titles and abstracts, followed by full-text evaluation to confirm eligibility. Discrepancies were resolved through consensus with a reviewer (A.Ö.). When numerical BDNF values were not reported, corresponding authors were contacted for additional data.

### 3.3. Participants’ and Interventions’ Characteristics

The included RCTs comprised 850 participants (407 in the control groups and 443 in the exercise groups). Participants’ ages ranged from 12 to 85 years. The majority of trials enrolled only female participants, while the rest included both sexes. In terms of diagnostic categories, three studies investigated individuals with multiple sclerosis [19,21,42], three studies involved patients with cognitive impairment or mild cognitive impairment [29,30,46], two studies included participants with depression [26,28], three studies targeted individuals with obesity or overweight [22,23,45], two studies was conducted in patients with type 2 diabetes mellitus [33,43], two studies enrolled patients with schizophrenia [31,32], 1 study focused on cancer survivors [24], 1 study focused on post-stroke hemiplegia [38], 1 study included participants with early subacute store [44], and 1 study included participants receiving hemodialysis [27].

Descriptive information about the studies included in the meta-analysis is presented in Table 3.

The included studies employed a wide range of physical exercise interventions, reflecting the diversity of modalities examined in relation to BDNF outcomes. Interventions comprised aerobic exercise (n = 7 studies [21,24,28,29,38,43,46]); resistance exercise (n = 3 studies [22,26,27]); combined programs (n = 9 studies [19,23,30,31,32,33,42,44,45]). Across the included studies, intervention durations were categorized as <8 weeks (n = 1), 8 weeks (n = 1), 12 weeks (n = 3), and 8–12 weeks (n = 14). Overall, most interventions were delivered over 8–12 weeks.

Specifically, nine interventions lasted 12 weeks; three lasted 16 weeks; three lasted 8 weeks; one lasted 26 weeks; one lasted 24 weeks; one lasted 10 weeks; and one study used a 3-week program. Session frequency ranged from 1 to 5 per week, with three sessions per week being the most common across studies. Session length ranged from approximately 20 min to 150 min, depending on the exercise modality. In most studies, control participants were instructed to maintain their habitual daily activities without exercise, and did not include sufficient information about the participants’ drug utilization status.

### 3.4. Statistical Analysis

In the analysis, random-effects models were used for all pooled effect-size calculations. Potential sources of heterogeneity were examined through subgroup analyses based on population type (neurological vs. non-neurological), assay type (serum vs. plasma), exercise type (aerobic, combined, resistance), and intervention duration categories (<8 weeks, 8 weeks, 8–12 weeks, and >12 weeks).

In addition, exploratory multivariate meta-regression models were conducted with the following moderators: intervention duration (weeks), weekly exercise frequency, minutes per session, total weekly minutes, and participant age. Small-study effects were evaluated through funnel plot inspection, Begg’s test, Egger’s regression test, and trim-and-fill analysis. All analyses were performed in R (v.4.4.2) using the metafor and meta packages, with *p* < 0.05 considered statistically significant.

## 4. Results

The analysis is based on 19 studies. Heterogeneity among the individual studies identified in this study was assessed using Cochran’s Q statistic and the I^2^ index to investigate inconsistencies in the studies’ results. In the study, the effect size index was calculated as the standardized difference between the averages. Cochran’s Q test yielded a value of 135.16 (df = 18, *p* < 0.0001), indicating statistically significant heterogeneity. The I^2^ statistic of 86.7% suggests substantial heterogeneity among the included studies, exceeding the standard threshold of 75%.

Additionally, the τ^2^ value of 0.89 (95% CI: 0.45–2.26) confirms significant between-study variance, as the confidence interval does not include zero. Given this substantial heterogeneity, a random-effects model was employed to estimate the pooled effect size.

**Figure 2:**
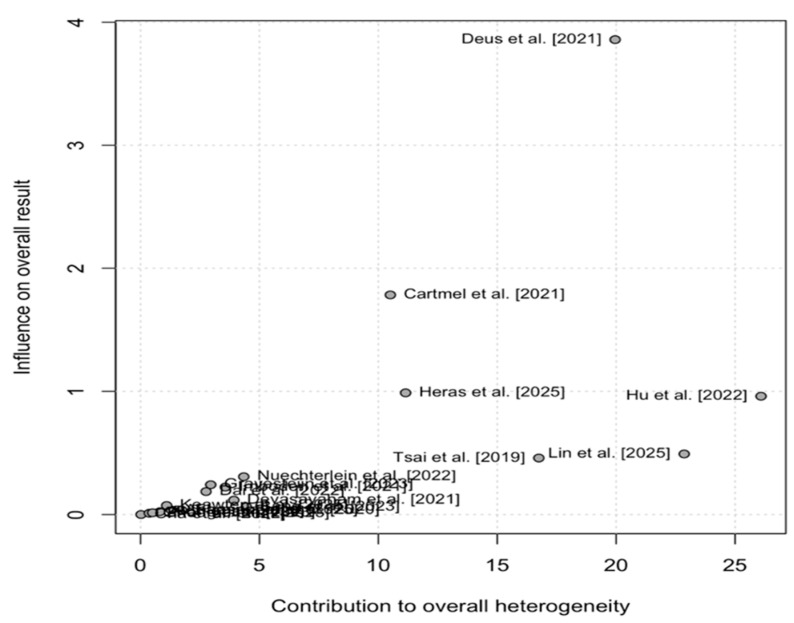
Baujat Plot (studies’ contributions to overall heterogeneity) [19,21,22,23,24,26,27,28,29,30,31,32,33,38,42,43,44,45,46].

The Baujat plots evaluate individual studies’ contributions to overall heterogeneity (*x*-axis: 0–25) and influence on the pooled effect size (*y*-axis: 0–4) in the meta-analysis. Labeled points across both visualizations, such as Cartmel et al. [24], Heras et al. [44], Nuechterlein et al. [32], and Lin et al. [46], reveal Deus et al. [27] as a prominent outlier in the upper-right quadrant, substantially driving both heterogeneity and effect variability, while most studies cluster in the low-left region with negligible impact. Cartmel et al. [24] exhibit moderate influence but low heterogeneity, underscoring that a minority of studies account for most of the dispersion (Figure 2).

**Figure 3:**
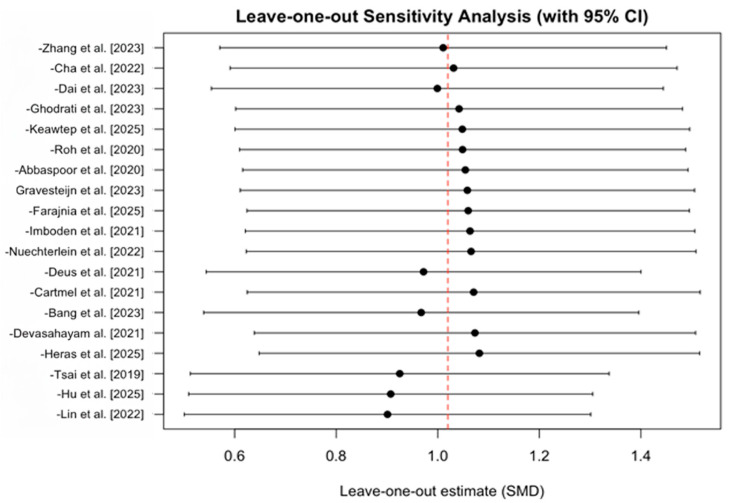
Leave-one-out Sensitivity Analysis [19,21,22,23,24,26,27,28,29,30,31,32,33,38,42,43,44,45,46].

The leave-one-out sensitivity analysis demonstrated that the overall pooled effect was highly stable, with the removal of any single study resulting in only minor changes to the summary estimate. The largest shifts were observed when excluding Lin et al. [46], Hu et al. [38], and Tsai et al. [29], which altered the pooled SMD by 0.119, 0.113, and 0.095, respectively; however, none of these exclusions moved the effect estimate outside the original confidence interval. All other studies [19,21,22,23,24,26,27,28,30,31,32,33,42,43,44,45] produced even smaller deviations (Figure 3).

**Figure 4:**
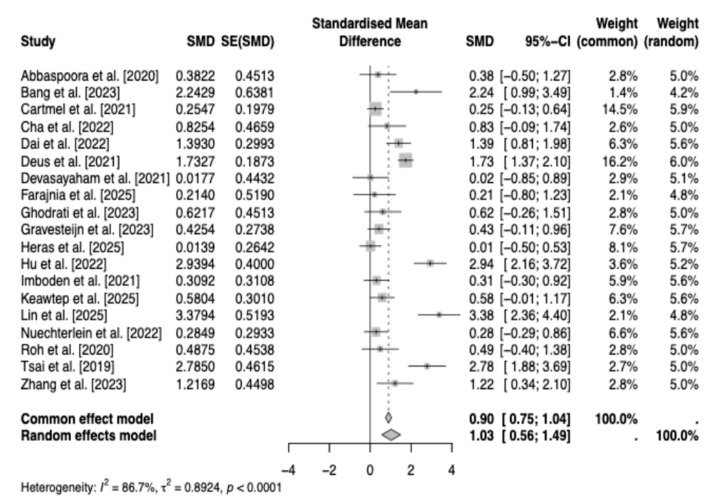
Forest Plot of Pooled Effect Sizes and Study Weights [19,21,22,23,24,26,27,28,29,30,31,32,33,38,42,43,44,45,46].

The meta-analysis included 19 studies, and the individual effect sizes are visualized in Figure 4.

A random-effects model was employed to pool standardized mean differences across studies [19,21,22,23,24,26,27,28,29,30,31,32,33,38,42,43,44,45,46], revealing a significant overall effect of exercise interventions on peripheral BDNF levels (SMD = 1.03, 95% CI [0.56, 1.49], z = 4.61, *p* < 0.0001). This corresponds to a large positive effect (>0.80 according to Cohen’s criteria), particularly evident in serum BDNF concentrations, with low-quality evidence. Substantial heterogeneity was present among the included studies (Q(18) = 135.16, *p* < 0.0001; I^2^ = 86.68%; τ^2^ = 0.7259), indicating that the variability in effect sizes substantially exceeds what would be expected from sampling error alone.

**Figure 5:**
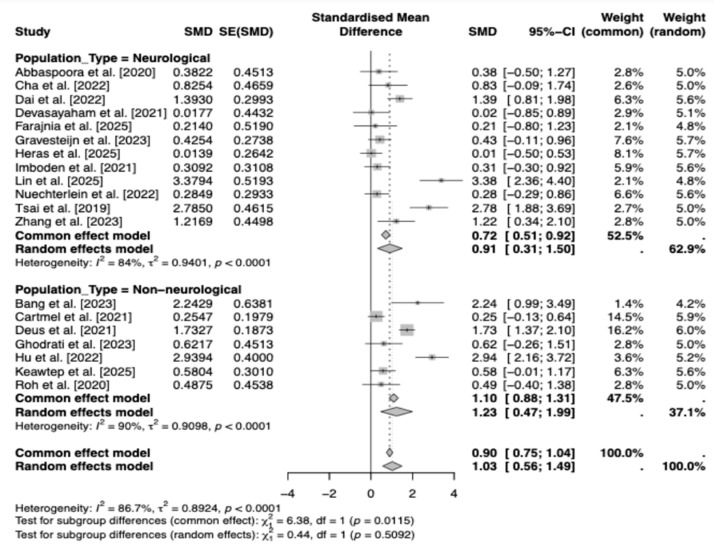
Forest Plot of Subgroup Analyses by Population Type [19,21,22,23,24,26,27,28,29,30,31,32,33,38,42,43,44,45,46].

The subgroup meta-analysis by population type (neurological vs. non-neurological) showed a large and significant overall effect of exercise on BDNF levels (SMD = 1.03, 95% CI [0.56, 1.49], z = 4.36, *p* < 0.0001), accompanied by substantial heterogeneity (τ^2^ = 0.89, I^2^ = 86.7%, Q(18) = 135.16, *p* < 0.0001) and it is essential to note that the certainty of evidence was low according to GRADE, primarily due to high heterogeneity and risk of bias in the included trials. Subgroup comparisons indicated no significant difference between neurological and non-neurological samples (Q(1) = 0.44, *p* = 0.51). Effect sizes were similar across groups—neurological (k = 12, SMD = 0.91, 95% CI [0.31, 1.50]) and non-neurological (k = 7, SMD = 1.23, 95% CI [0.47, 1.99])—though wide confidence intervals suggest imprecision and highlight the need for larger studies (Figure 5).

**Figure 6:**
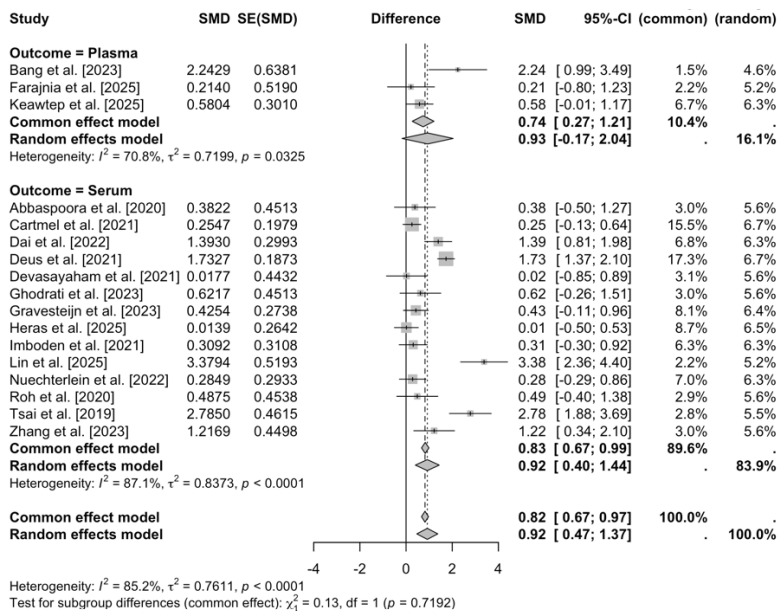
Forest plot of Subgroup Analyses by Assay Type [19,21,22,23,24,27,28,29,30,31,32,33,42,43,44,45,46].

Excluding two studies [26,38] due to unavailable serum or plasma BDNF data, the subgroup meta-analysis of the remaining 17 studies (k = 17) [19,21,22,23,24,27,28,29,30,31,32,33,42,43,44,45,46], by assay type (plasma vs. serum) demonstrated a large, significant overall positive effect of exercise on BDNF levels (SMD = 0.92, 95% CI [0.47, 1.37], z = 3.97, *p* < 0.0001), with persistent high heterogeneity (τ^2^ = 0.76 [95% CI: 0.36, 2.17], I^2^ = 85.2% [77.7%, 90.2%], Q(16) = 108.11, *p* < 0.0001; H = 2.60). Subgroup estimates were comparable: plasma (k = 3; SMD = 0.93, 95% CI [−0.17, 2.04], τ^2^ = 0.72) and serum (k = 14; SMD = 0.92, 95% CI [0.40, 1.44], τ^2^ = 0.84), with no significant differences between groups in random-effects models (Q(1) = 0.00, *p* = 0.98). These findings suggest assay type does not moderate the BDNF response to exercise, inconclusively though the wide CIs for plasma (limited k) and unresolved heterogeneity (I^2^ = 85.2%) indicate the need for larger studies to confirm consistency across measurement modalities (Figure 6).

**Figure 7:**
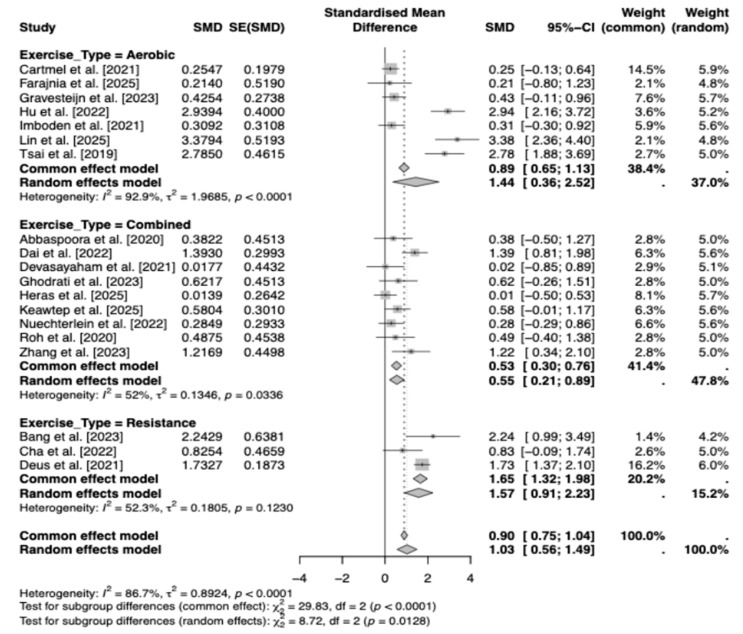
Forest plot of Subgroup Analyses by Exercise Type [19,21,22,23,24,26,27,28,29,30,31,32,33,38,42,43,44,45,46].

The subgroup meta-analysis by exercise type (aerobic, combined, resistance; k = 19 studies) [19,21,22,23,24,26,27,28,29,30,31,32,33,38,42,43,44,45,46] yielded a large, significant overall positive effect of exercise on BDNF levels (SMD = 1.03, 95% CI [0.56, 1.49], z = 4.36, *p* < 0.0001). Our findings should be interpreted as exploratory and hypothesis-generating. Because of the low GRADE certainty rating and tempered by high heterogeneity (τ^2^ = 0.89 [95% CI: 0.45, 2.26], I^2^ = 86.7% [80.6%, 90.9%]). Subgroup effects were most pronounced for resistance training [22,26,27] (k = 3; SMD = 1.57, 95% CI [0.91, 2.23], τ^2^ = 0.18), followed by aerobic [21,24,28,29,38,43,46] (k = 7; SMD = 1.44, 95% CI [0.36, 2.52], τ^2^ = 1.97) and combined interventions [19,23,30,31,32,33,42,44,45] (k = 9; SMD = 0.55, 95% CI [0.21, 0.89], τ^2^ = 0.13), with significant between-subgroup differences (Q(2) = 8.72, *p* = 0.013). These patterns suggest that resistance and aerobic exercises may elicit stronger BDNF responses than combined protocols, although wide CIs (particularly for aerobic) and elevated τ^2^ underscore unresolved variability, calling for more homogeneous trials to clarify type-specific benefits (Figure 7).

**Figure 8:**
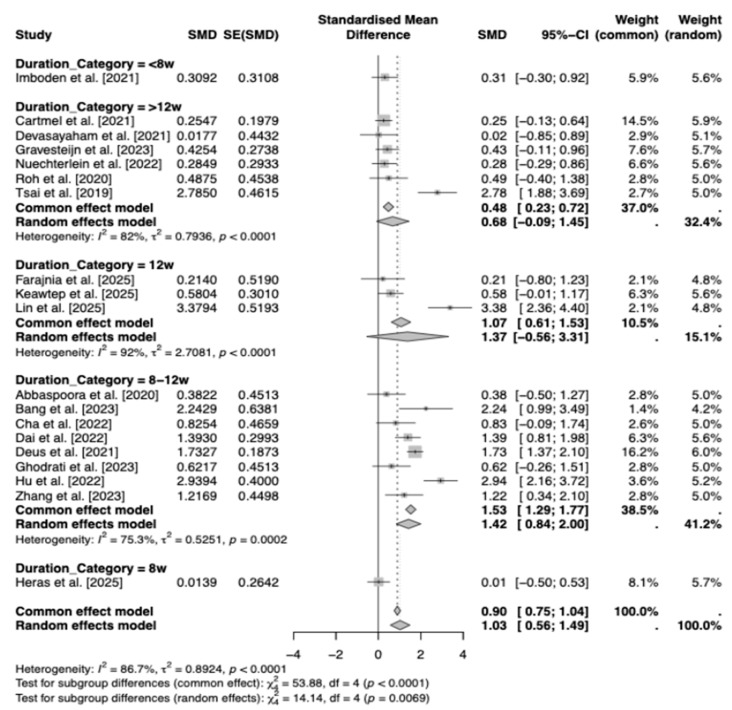
Forest plot of Subgroup Analyses by Duration [19,21,22,23,24,26,27,28,29,30,31,32,33,38,42,43,44,45,46].

According to low quality evidence, the subgroup meta-analysis by intervention duration category revealed a large, significant overall positive effect of exercise on BDNF levels (SMD = 1.03, 95% CI [0.57, 1.49], z = 4.36, *p* < 0.0001), accompanied by substantial heterogeneity (τ^2^ = 0.89 [95% CI: 0.45, 2.26], I^2^ = 86.7% [80.6%, 90.9%], H = 2.74 [2.27, 3.31]; Q(18) = 135.16, *p* < 0.0001; inverse-variance method, REML τ^2^ estimator, Q-profile CIs). Subgroup effects were strongest for 8–12 weeks [19,22,26,27,30,31,33,38] (k = 8; SMD = 1.42, 95% CI [0.84, 2.00], τ^2^ = 0.53) and 12 weeks [43,45,46] (k = 3; SMD = 1.37, 95% CI [−0.56, 3.31], τ^2^ = 2.71), moderate for >12 weeks [21,23,24,29,32,42] (k = 6; SMD = 0.68, 95% CI [−0.09, 1.45], τ^2^ = 0.79), and negligible for single-study categories (<8 weeks: SMD = 0.31, 95% CI [−0.30, 0.92] [28]; 8 weeks: SMD = 0.01, 95% CI [−0.50, 0.53]) [44]. Significant between-subgroup differences emerged (Q(4) = 14.14, *p* = 0.007), suggesting that shorter-to-moderate durations optimize BDNF gains. However, wide CIs and elevated τ^2^ (particularly at 12 weeks) underscore unresolved variability, necessitating larger trials to refine protocols (Figure 8).

The multivariate meta-regression model examined the influence of continuous moderators (duration (weeks), frequency per week, minutes per session, total weekly minutes, participant age, and baseline BDNF levels) on the pooled effect size of exercise interventions on BDNF levels. The intercept (β = 1.0484, SE = 0.1971, z = 5.3205, *p* < 0.0001, 95% CI [0.6622, 1.4346]) represents the estimated average effect when all moderators are at their mean values, indicating a robust positive overall effect of exercise on BDNF. Age emerged as a significant predictor (β = 0.0298, SE = 0.0134, z = 2.2284, *p* = 0.0259, 95% CI [0.0036, 0.0559]), suggesting that the magnitude of exercise-induced BDNF increases tends to rise modestly with participant age. Baseline BDNF level was also a significant moderator (β = −0.0395, SE = 0.0177, z = −2.2297, *p* = 0.0258, 95% CI [−0.0741, −0.0048]), indicating that individuals with higher baseline BDNF levels exhibit attenuated exercise responses. In contrast, duration (β = 0.0611, *p* = 0.2040), frequency (β = 0.3409, *p* = 0.2461), minutes per session (β = −0.0127, *p* = 0.1565), and total weekly minutes (β = −0.0003, *p* = 0.9423) showed nonsignificant associations, with confidence intervals spanning zero and effect estimates close to null, suggesting minimal influence on the outcome. Collectively, the moderators accounted for a moderate proportion of the observed heterogeneity (R^2^ = 35.07%) (Table 4).

**Figure 9:**
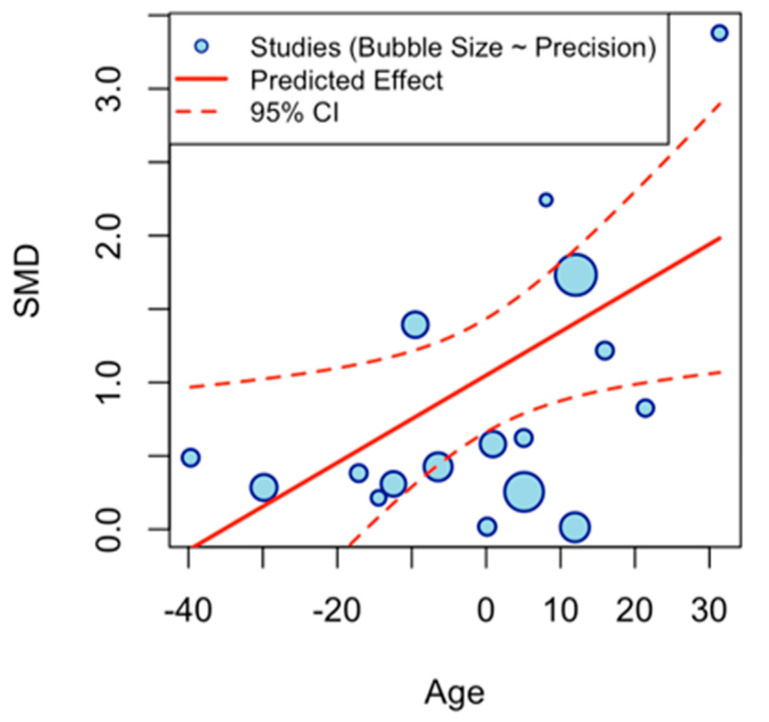
Scatter Plot of Age Moderator.

A scatter plot illustrates the meta-regression of age on the effect size for BDNF. The *x*-axis shows centered age values (−40 to 30), and the *y*-axis depicts g (0 to 3.5). Blue points represent individual studies, bubble size indicates study precision, with a red dashed line indicating a positive slope. The plot reveals a modest positive association: effect sizes increase slightly with age, suggesting greater BDNF benefits in older participants. However, point dispersion highlights residual heterogeneity, implying other factors influence the relationship (Figure 9).

**Figure 10:**
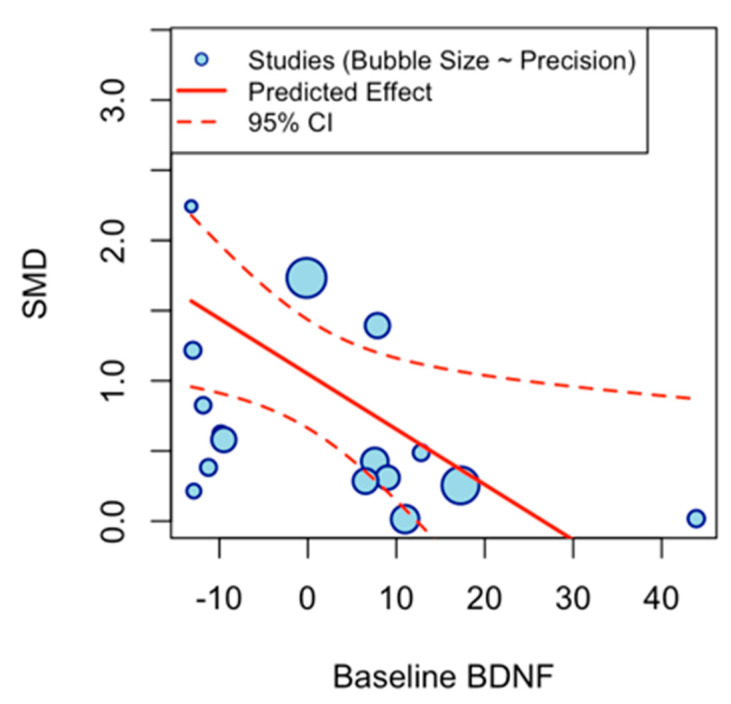
Scatter Plot of Baseline BDNF Level Moderator.

A scatter plot illustrates the meta-regression of age on the effect size for BDNF. The *x*-axis shows centered age values (−10 to 40), and the *y*-axis depicts g (0 to 3.5). The plot reveals a modest negative association: higher baseline BDNF levels were associated with attenuated effects. However, point dispersion highlights residual heterogeneity, implying other factors influence the relationship (Figure 10).

**Figure 11:**
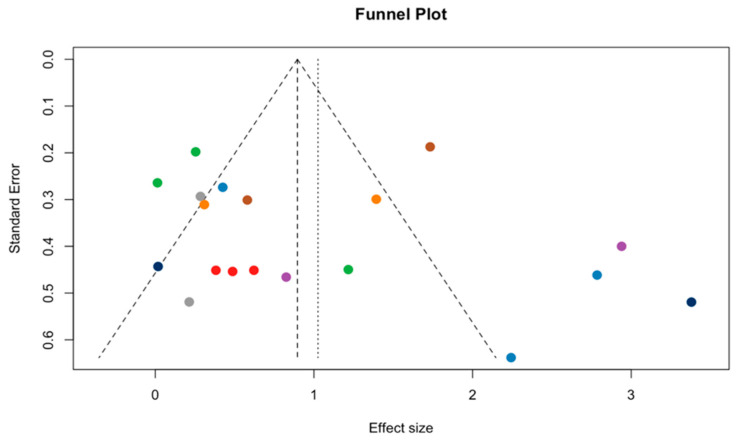
Funnel plot assessing publication bias. Each color represents a different study.

The funnel plot visualizes potential publication bias in the meta-analysis of exercise effects on BDNF, plotting study effect sizes on the *x*-axis (0–3) against standard errors on the *y*-axis (0–0.06). The expected inverted-funnel pattern is largely apparent: larger studies (lower SE) cluster near the pooled estimate (SMD ≈ 1.0) at the bottom of the plot, while smaller studies (higher SE) are more widely dispersed toward the top. There is slight left-side sparsity—fewer studies reporting near-null or negative effects—which may indicate some underreporting of small, non-significant studies and a potential upward bias in the pooled estimate. However, formal tests do not support substantial small-study bias: Begg’s rank correlation test was non-significant (*p* = 0.068), and Egger’s regression intercept was also non-significant (*p* = 0.113). Trim-and-fill procedures imputed no missing studies. Robustness checks indicated a large classic fail-safe N (≈994), although Orwin’s fail-safe N for a trivial effect (g = 0.20) was relatively small (4). Taken together, these findings suggest a low-to-moderate risk of publication bias (Figure 11).

## 5. Discussion

This meta-analysis demonstrates that exercise programs are associated with significant increases in peripheral (serum/plasma) BDNF across clinical populations, indicating a positive effect under a random-effects model (DerSimonian-Laird) with low-quality evidence. Between-study heterogeneity was very high, necessitating cautious interpretation and motivating exploration of potential moderators. Multivariate meta-regression with continuous moderators (duration in weeks, weekly frequency, minutes per session, total weekly minutes, and participant age) revealed a significant age effect, where effect sizes modestly increased with advancing age, potentially reflecting heightened BDNF responsiveness in older adults, and higher baseline BDNF levels were associated with attenuated effects. Other moderators were not statistically significant, collectively accounting for limited heterogeneity. A scatter plot of age versus effect size (Figure 9) showed a modest positive association (red dashed line), with blue points (studies) indicating slight increases alongside residual dispersion, suggesting the presence of other unmodeled factors. Subgroup analyses further illuminated variability: population type showed comparable effects for neurological and non-neurological conditions, suggesting BDNF gains may not be disease-specific. Excluding two studies [26,38] due to the absence of serum/plasma data, the outcome subgroups yielded similar effects for plasma and serum; however, the small number of plasma-based studies limits firm conclusions about assay-type differences. Subgroup analyses showed that resistance and aerobic training tended to elicit larger increases in BDNF than combined interventions. Exercise type subgroups highlighted resistance and aerobic as superior to combined interventions. However, these subgroup effects are exploratory and statistically uncertain due to the low-quality evidence. Similarly, shorter-to-moderate interventions (8–12 weeks) appeared more effective than longer ones, although wide confidence intervals indicate that these patterns require confirmation in larger, well-powered trials.

To our knowledge, this study presents the first comprehensive synthesis in the literature that simultaneously compares neurological and non-neurological patient groups within a single meta-analytic model, thereby offering an integrated evaluation of the relative influence of moderators, including baseline BDNF level, exercise duration, age, and exercise type, on exercise-induced BDNF responses. The results also revealed substantial heterogeneity (I^2^ ≈ 86%; τ^2^ = 0.42). This level of variability suggests that methodological differences—including exercise type, duration, intensity, and measurement timing—as well as sample diversity may contribute to fluctuations in effect size. Therefore, although the overall effect of exercise on BDNF is robust, generalizations about its magnitude and consistency should be made cautiously.

This pattern is consistently supported by studies showing that exercise enhances both BDNF levels and cognitive function in middle-aged and older adults [47,48,49]. Mechanistically, age-related alterations in BDNF–TrkB (Tropomyosin-related receptor kinase B) signaling have been reported to modulate neuroplastic responses to physical activity [50]. In response to exercise, Fibronectin Domain Containing Protein-5 (FNDC5) activates Peroxisome Proliferator Activated Receptor Gamma Coactivator 1-Alpha (PGC-1α), a metabolic mediator that enhances BDNF level in brain neurons. The hippocampus produces BDNF when FNDC5 is delivered peripherally using adenoviral vectors [51]. The rise in BDNF levels activates the TrkB receptor and its downstream signaling [52]. Moreover, even in neurodegenerative conditions, exercise has been shown to elevate BDNF levels [53]. Collectively, these findings indicate that exercise is a potent environmental regulator that supports BDNF-mediated neuroplasticity despite aging. On the other hand, parameters such as intervention duration, session frequency, session length, and total weekly exercise time were not significantly associated with the BDNF response. This finding suggests that exercise-induced neurotrophic adaptations may not follow a strict dose–response curve and that comparable physiological effects can be achieved with varying protocols. Indeed, meta-analyses examining both acute and chronic exercise [35,54] likewise failed to identify a consistent association between exercise variables and increases in BDNF.

Nonlinear or threshold-dependent patterns have also been observed for other exercise-induced factors. Insulin-like growth factor-1 (IGF-1) exhibits a curvilinear response to training load, with moderate exercise producing the greatest increases and excessive volume attenuating them [55]. Resistance training may further enhance neuroplasticity via IGF-1–mediated pathways [56]. Likewise, vascular endothelial growth factor (VEGF) expression varies by fiber type and intensity, peaking in fast-twitch fibers at moderate-to-high workloads [57]. Together, these findings suggest that neurotrophic and angiogenic responses may be most significant within a moderate stimulus range, though this remains exploratory.

When considering moderator analyses, exercise type appeared to explain some variability in BDNF responsiveness; however, this observation is exploratory and should not be interpreted as a reliable predictor, given residual heterogeneity and small subgroup sizes. Duration and frequency alone did not account for the observed variability. Subgroup analyses indicated that the only program variable that appeared to differentiate the BDNF response was exercise type. Resistance training produced the strongest increase, followed by aerobic and combined protocols. This finding aligns with recent meta-analyses reporting pronounced neurotrophic effects of resistance training, particularly in adult and older populations [58,59]. However, some studies suggest that aerobic or combined aerobic and resistance protocols may be more effective, particularly in older individuals [15,60,61]. Such discrepancies imply that the relative impact of exercise modalities on BDNF is sensitive to factors such as age, population characteristics, cognitive status, and intervention design. For instance, aerobic exercise produces more consistent increases in adolescents [62], whereas in older adults with cognitive impairment, aerobic and resistance training can yield comparable neurotrophic responses through distinct mechanisms [29]. Thus, although the ranking observed in our study aligns with the heterogeneous trends in the literature, more standardized protocols are needed to establish this relationship with greater certainty.

Resistance exercise has been proposed as a potent neurotrophic stimulus that may enhance BDNF synthesis through mechanical tension and intramuscular contractile signaling [63,64]. Lactate—a metabolite released during muscular activity that crosses the blood–brain barrier—has also been shown to upregulate BDNF through activation of the Silent Information Regulator 1 (SIRT1)–Peroxisome Proliferator-Activated Receptor Gamma Coactivator 1-Alpha (PGC-1α)–Fibronectin Type III Domain-Containing Protein 5 (FNDC5) pathway, indicating that metabolic stress may play a key role in neuroplastic adaptations [65,66]. This mechanism may further involve Hydroxycarboxylic Acid Receptor 1 (HCAR1)–mediated activation of the Extracellular Signal-Regulated Kinase (ERK)– cAMP Response Element-Binding Protein (CREB) cascade, which regulates plasticity-related genes such as BDNF and VGF (nerve growth factor-inducible) [67]. However, as these mechanistic pathways were not directly examined in the present meta-analysis, they should be interpreted as physiological inferences rather than causal conclusions.

With respect to combined exercise, some evidence suggests that endurance-based stimuli may attenuate resistance-specific anabolic signaling via Adenosine Monophosphate-activated Protein Kinase (AMPK), thereby producing an ‘interference’ effect in combined protocols [68]. In this context, the more limited increases in BDNF observed with combined exercise in our study may reflect mechanical and metabolic interactions inherent to the sequential application of resistance and aerobic loads, which may constrain the neurotrophic response.

In interpreting these findings, it is essential to consider the biological origin of circulating BDNF. BDNF is predominantly stored in platelets, and serum levels are strongly influenced by platelet degranulation during coagulation. As a result, serum BDNF concentrations are overwhelmingly reflective of platelet-derived pools rather than direct secretion from neural tissue [69]. This has important implications for interpretation, particularly in mechanistic contexts, as peripheral BDNF levels may conflate signals from distinct biological sources. Nonetheless, evidence suggests that circulating BDNF arises from multiple compartments, including neural, muscular, and hematologic sources [70]. Arterial–venous sampling studies have indicated that the brain itself may contribute meaningfully to circulating BDNF during exercise, potentially accounting for up to 70–80% of systemic levels under specific physiological conditions [57]. This possibility is further supported by findings that endurance training may enhance resting BDNF release in the brain [71]. Additionally, skeletal muscle has been shown to express and release BDNF in response to contraction, suggesting a peripheral source that may also modulate systemic levels [70]. Given this complexity, serum BDNF should be interpreted with caution, as the relative contributions of central, muscular, and platelet sources likely vary with the metabolic, neural, and hemodynamic context in which it is measured.

The emergence of age as a significant moderator aligns with existing physiological frameworks, suggesting that age-related reductions in muscle mass, anabolic resistance, and basal BDNF/IGF-1 levels may enhance neurotrophic sensitivity to resistance exercise [72]. In our subgroup analysis, resistance and aerobic training appeared to produce greater BDNF increases than combined interventions, with a statistically significant difference between modalities. This pattern may reflect biologically plausible differences in neurotrophic responsiveness across exercise types [73].

In addition, higher baseline BDNF concentrations were linked to smaller exercise-induced increases, suggesting a potential ceiling effect. Individuals with lower initial BDNF may therefore exhibit greater neurotrophic adaptation to training. This observation is consistent with prior evidence that individuals with higher basal BDNF levels or greater training status exhibit attenuated neurotrophic responses to exercise [74]. However, given the limited number of studies reporting baseline levels and the exploratory nature of this association, this finding should be interpreted cautiously and verified in larger, standardized trials.

The subgroup analysis examining the impact of intervention duration on the BDNF response showed that 8–12-week protocols produced more pronounced increases. Our findings are consistent with recent meta-analyses reporting that 8–12 weeks represent the most effective window for eliciting neurotrophic adaptations [54,61,75]. Nonetheless, the wide confidence intervals around the 12-week mark indicate that duration-dependent thresholds have not yet been firmly established. More clearly delineating this relationship will require large sample, standardized, randomized controlled trials.

The subgroup analysis comparing serum and plasma indicated no statistically significant difference in the magnitude of exercise-induced changes in BDNF. Similar results have been reported in older adult populations [61], in which both serum and plasma concentrations increased following training, yet heterogeneity remained unresolved. Given the limited number of plasma-based studies, the current evidence remains inconclusive. Serum BDNF primarily originates from platelets, whereas plasma reflects circulating, non-platelet BDNF [69]. Therefore, these results should not be interpreted as confirmatory.

In the final subgroup analysis, no significant difference was observed between individuals with and without neurological disorders regarding exercise-induced increases in BDNF. This finding—showing that neurological conditions do not markedly diminish the neurotrophic effect of exercise—corresponds with studies reporting that post-exercise elevations in BDNF are independent of disease status across different neurological conditions [54,75,76,77]. However, because the available evidence is derived from relatively small samples, caution is warranted when generalizing these findings to broader neurological populations.

Considered as a whole, the present meta-analysis constitutes a novel examination by evaluating both neurological and non-neurological individuals within a single meta-analytic model, thereby providing a multidimensional assessment of exercise effects on BDNF. In this respect, it strengthens the proposition that exercise may serve as a supportive intervention for central nervous system function regardless of diagnosis. Given the findings, resistance training may be incorporated as a core component, and aerobic exercise as a complementary element, when designing exercise programs for adults and older adults. From a duration perspective, interventions lasting 8–12 weeks appear to represent the most effective window for eliciting BDNF responses.

Although this study presents a comprehensive analysis, several limitations should be acknowledged. First, substantial methodological and design-related variability across the included studies resulted in high heterogeneity; differences in ages, exercise modality, duration, intensity, sample characteristics, and BDNF sampling timelines complicate the interpretation of pooled effect sizes. Small subgroup sizes further contribute to uncertainty regarding moderators such as age, clinical diagnosis, and intervention duration. Additionally, the uneven distribution of serum- versus plasma-based assays limited interpretability in matrix comparisons. Second, blinding of participants, therapists, and outcome assessors was rarely implemented, and most studies employed relatively small sample sizes, both of which constrain the methodological robustness and generalizability of the pooled estimates. Third, although the overall methodological quality of the included studies was generally high, several trials were rated as only “fair” (PEDro score = 5), introducing a potential risk of bias. To evaluate the impact of these studies, we conducted a sensitivity analysis excluding all trials with PEDro ≤ 5. The pooled effect size, confidence intervals, and direction of results remained essentially unchanged. This indicates that lower-quality studies did not materially drive the primary conclusions; however, they should still be interpreted with appropriate caution. Future meta-analyses supported by larger, high-quality RCTs with more rigorous blinding will help strengthen the certainty of evidence.

Overall, exercise interventions significantly boost peripheral BDNF in clinical populations, supporting the biological plausibility of exercise-induced neuroplasticity. However, the high heterogeneity across studies underscores the need to systematically explore moderators in future research. Special focus should be on refining exercise prescriptions by type and intensity, backed by standardized biomarker protocols and sufficiently powered trials, to identify which interventions most effectively increase BDNF in specific patient groups. Future studies should also adopt open science practices to improve transparency and reproducibility. This includes pre-registering hypotheses and analysis plans, sharing data, utilizing harmonized biomarker protocols for BDNF measurement, and standardizing exercise reporting according to FITT principles. These efforts will help reduce reporting bias and enable more accurate, comparable meta-analyses across diverse clinical populations.

## 6. Conclusions

Based on low-quality evidence, this meta-analysis indicates that exercise interventions may increase peripheral BDNF across clinical populations, reinforcing the role of physical activity as a potent modulator of neuroplasticity. Exercise type, particularly resistance and aerobic protocols, appeared to elicit larger BDNF increases than combined interventions. However, these findings are exploratory and should be interpreted cautiously. In contrast, duration and frequency alone were insufficient to explain the variability. In light of the exploratory findings and low certainty of evidence, appropriately prescribed exercise, particularly resistance and aerobic interventions, may offer neurotrophic benefits with potential cognitive and clinical implications across various conditions. The observed heterogeneity may stem from variations in samples and methodological choices; therefore, adopting more consistent methodological approaches in future biomarker assessments is of considerable importance. Future trials should adopt harmonized FITT-based reporting, incorporate pre-registered moderator analyses, and clarify how exercise modality, type, and duration can be optimized to maximize neuroplastic and therapeutic effects. In clinical practice, integrating tailored multimodal exercise programs may represent a cost-effective, non-pharmacological strategy to enhance brain health and support public health initiatives. To strengthen evidence certainty, upcoming trials should ensure sufficient statistical power, allocation concealment, and blinding of assessors.

## Figures and Tables

**Table 1:** Quality of Included Trials Based on the Physiotherapy Evidence Database (PEDro) Criteria and Scores.

Study	EC	I	II	III	IV	V	VI	VII	VIII	IX	X	Total	Quality Level
Abbaspoor et al. (2020) [19]	Y	✓	✓	✓	0	0	✓	0	✓	✓	✓	8	Good
Bang (2023) [22]	Y	✓	0	✓	0	0	✓	0	✓	✓	✓	7	Good
Cartmel et al. (2021) [24]	Y	✓	0	✓	0	0	0	0	0	✓	✓	5	Fair
Cha et al. (2022) [26]	Y	✓	✓	✓	0	0	✓	0	✓	✓	✓	8	Good
Dai et al. (2022) [31]	Y	✓	✓	✓	✓	0	✓	0	✓	✓	✓	9	Excellent
Deus et al. (2021) [27]	Y	✓	0	✓	0	0	0	0	0	✓	✓	5	Fair
Devasahayam et al. (2021) [42]	Y	✓	✓	✓	0	0	✓	✓	✓	✓	✓	9	Excellent
Farajnia et al. (2025) [43]	Y	✓	0	0	0	0	✓	✓	0	✓	✓	6	Good
Ghodrati et al. (2023) [33]	Y	✓	0	✓	0	0	✓	0	0	✓	✓	6	Good
Gravesteijn et al. (2023) [21]	Y	✓	✓	✓	0	0	0	✓	0	✓	✓	7	Good
De Las Heras et al. (2025) [44]	Y	✓	0	✓	0	0	0	✓	✓	✓	✓	7	Good
Hu et al. (2022) [38]	Y	✓	0	✓	0	0	0	0	0	✓	✓	5	Fair
Imboden et al. (2021) [28]	Y	✓	✓	✓	0	0	✓	0	✓	✓	✓	8	Good
Keawtep et al. (2024) [45]	Y	✓	✓	✓	0	0	✓	✓	✓	✓	✓	9	Excellent
Lin et al. (2025) [46]	Y	✓	✓	✓	✓	✓	✓	✓	0	✓	✓	10	Excellent
Nuechterlein et al. (2023) [32]	Y	✓	✓	✓	0	0	✓	0	0	✓	✓	7	Good
Roh et al. (2020) [23]	Y	✓	0	0	0	0	0	0	✓	✓	✓	5	Fair
Tsai et al. (2019) [29]	Y	✓	✓	✓	0	0	✓	0	✓	✓	✓	8	Good
Zhang et al. (2023) [30]	Y	✓	✓	✓	0	0	✓	✓	✓	✓	✓	9	Excellent
Total												7.2	Good

EC: eligibility criteria; Y: Yes; I: random allocation; II: concealed allocation; III: baseline comparability; IV: blinding of subjects; V: blinding of researchers/evaluators; VI: blinding of assessors; VII: measure of at least one key outcome obtained from more than 85% of subjects initially allocated to groups; VIII: intention to treat; IX: comparison results between groups; X: measured at least one key outcome at two time points; ✓, criterion is present; 0, criterion is missing.

**Table 2:** Quality of Evidence GRADE Assessment.

Certainty Assessment							Number of Patients		Effect	Certainty	Importance
Number of Studies	Study Design	Risk of Bias	Inconsistency	Indirectness	Imprecision	Other Considerations	Exercise	Control	Absolute (95% CI)		
19	Randomised trials	Serious ^a^	Serious ^b^	Not serious	Not serious	None	443	407	SMD: 1.03 (0.56–1.49)	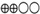 Low	IMPORTANT

Notes: ^a^ 17 studies (89.5%) did not clarify whether the subjects were blinded to the intervention, and only 1 study presented information on the blinding of researchers; ^b^ Substantial heterogeneity (I^2^ > 50%); **CI**: confidence interval; **SMD**: Standardized Mean Difference. 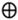: Certainty Level.

**Table 3:** Descriptive Information Related to the Studies.

Study	Sample Size	Clinical Condition	Age	Exercise Type	Duration (Weeks)	Frequency (Sessions/Week)	Session Length (min)	BDNF Outcome (Serum/Plasma)	Drug Utilization	Effect Size (Hedge’s g)
Abbaspoor et al. (2020) [19]	Exp. (n = 10) Ctrl. (n = 10)	Multiple sclerosis	Exp. 33.50 ± 6.37 Ctrl. 36.75 ± 6.80	Combined	8	3	37.5	BDNF ↔ (Serum)	UN	0.38
Bang et al. (2023) [22]	Exp. (n = 8) Ctrl. (n = 8)	Obesity	Exp. 60.00 ± 2.97 Ctrl. 60.62 ± 2.77	Resistance	12	3	60	BDNF ↑ (Plasma)	No	2.24
Cartmel et al. (2021) [24]	Exp. (n = 52) Ctrl. (n = 51)	Ovarian cancer	Exp. 57.30 ± 8.80 Ctrl. 57.40 ± 8.50	Aerobic	26	4	150	BDNF ↔ (Serum)	UN	0.25
Cha et al. (2022) [26]	Exp. (n = 10) Ctrl. (n = 10)	Depression	Exp. 74.80 ± 6.76 Ctrl. 72.50 ± 6.52	Combined	12	3	70	BDNF ↑ (Serum)	UN	0.83
Dai et al. (2022) [31]	Exp. (n = 25) Ctrl. (n = 31)	Schizophrenia	Exp. 41.40 ± 7.86 Ctrl. 44.06 ± 8.40	Combined	12	3	60	BDNF ↑ (Serum)	Yes	1.39
Deus et al. (2021) [27]	Exp. (n = 81) Ctrl. (n = 76)	Depression	Exp. 62.27 ± 3.24 Ctrl. 66.33 ± 3.88	Resistance	12	3	30	BDNF ↑ (Serum)	UN	1.73
Devasahayam et al. (2021) [42]	Exp. (n = 14) Control (n = 8)	Multiple sclerosis	Exp. 54.07 ± 8.46 Ctrl. 50.71 ± 12.08	Combined	24	3	60	BDNF ↔ (Serum)	UN	0.02
Farajnia et al. (2025) [43]	Exp. (n = 7) Control (n = 8)	Multiple sclerosis	Exp. 34.30 ± 8.42 Ctrl. 41.30 ± 9.98	Aerobic	10	2	50	BDNF ↑ (Plasma)	No	0.21
Ghodrati et al. (2023) [33]	Exp. (n = 12) Ctrl. (n = 9)	Type 2 diabetes mellitus	Exp. 58.80 ± 1.50 Ctrl. 55.80 ± 1.50	Combined	12	3	65	BDNF ↔ (Serum)	No	0.62
Gravesteijn et al. (2023) [21]	Exp. (n = 30) Ctrl. (n = 25)	Multiple sclerosis	Exp. 43.50 ± 10.10 Ctrl. 48.10 ± 10.60	Aerobic	16	2	60	BDNF ↓ (Serum)	Yes	0.43
De Las Heras et al. (2025) [44]	Exp. (n = 38) Ctrl. (n = 23)	Early Subacute Stroke	Exp. 63.00 ± 11.39 Ctrl. 65.35 ± 8.68	Combined	8	3	20	BDNF ↔ (Serum)	UN	0.01
Hu et al. (2022) [38]	Exp. (n = 26) Ctrl. (n = 26)	Post-stroke hemiplegia	Exp. 56.22 ± 10.37 Ctrl. 56.97 ± 10.24	Aerobic	8	3	40	BDNF ↑ (UN)	UN	2.94
Imboden et al. (2021) [28]	Exp. (n = 22) Ctrl. (n = 20)	Depression	Exp. 41.30 ± 9.20 Ctrl. 38.30 ± 13.40	Aerobic	3	5	45	BDNF ↔ (Serum)	UN	0.31
Keawtep et al. (2024) [45]	Exp. (n = 23) Ctrl. (n = 23)	Obesity	Exp. 52.70 ± 3.60 Ctrl. 53.61 ± 2.81	Combined	12	1	60	BDNF ↑ (Plasma)	UN	0.58
Lin et al. (2025) [46]	Exp. (n = 18) Ctrl. (n = 18)	Mild cognitive dysfunction	Exp. 85.28 ± 4.65 Ctrl. 81.96 ± 6.12	Aerobic	12	3	30	BDNF ↑ (Serum)	UN	3.38
Nuechterlein et al. (2023) [32]	Exp. (n = 24) Ctrl. (n = 23)	Schizophrenia	Exp. 22.00 ± 3.30 Ctrl. 22.80 ± 4.60	Combined	12	4	45	BDNF ↔ (Serum)	UN	0.28
Roh et al. (2020) [23]	Exp. (n = 10) Ctrl. (n = 10)	Obesity	Exp. 12.60 ± 0.52 Ctrl 12.50 ± 0.53	Combined	16	5	60	BDNF ↑ (Serum)	UN	0.49
Tsai et al. (2019) [29]	Exp. (n = 19) Ctrl. (n = 18)	Cognitive impairment	Exp. 66.00 ± 7.68 Ctrl. 65.17 ± 7.00	Combined	16	3	40	BDNF ↑ (Serum)	No	2.78
Zhang et al. (2023) [30]	Exp. (n = 14) Ctrl. (n = 10)	Cognitive impairment	Exp. 66.67 ± 6.04 Ctrl. 69.75 ± 7.02	Combined	12	3	60	BDNF ↑ (Serum)	Yes	1.22

Exp.: Experiment; Ctrl.: Control; BDNF: Brain-Derived Neurotrophic Factor; ↑: Significant increase in BDNF levels; ↓: Significant decrease in BDNF levels; ↔: No improvement/change in BDNF levels; UN: Unknown.

**Table 4:** Multivariate Meta-Regression Coefficients: Influence of Moderators on Effect Size.

Moderator	Estimate	SE	z	*p*	CI 95%
Intercept	1.0484	0.1971	5.3205	<0.0001	[0.6622–1.4346]
Duration	0.0611	0.0481	1.2703	0.2040	[−0.0332–0.1555]
Frequency	0.3409	0.2939	1.1600	0.2461	[−0.2351–0.9169]
Minutes Per Session	−0.0127	0.0090	−1.4169	0.1565	[−0.0303–0.0049]
Total Weekly Minutes	−0.0003	0.0043	−0.0723	0.9423	[−0.0088–0.0081]
Age	0.0298	0.0134	2.2284	0.0259 *	[0.0036–0.0559]
Baseline BDNF Level	−0.0395	0.0177	−2.2297	0.0258 *	[−0.0741–−0.0048]

* *p* < 0.05.

## Data Availability

The original contributions presented in this study are included in the article/Supplementary Materials. Further inquiries can be directed to the corresponding authors.

## Supplementary Materials

The following supporting information can be downloaded at: https://www.mdpi.com/article/10.3390/brainsci16010039/s1. PRISMA 2020 Checklist. Ref. [78] was cited in supplementary materials.
